# Is regular drinking in later life an indicator of good health? Evidence from the English Longitudinal Study of Ageing

**DOI:** 10.1136/jech-2015-206949

**Published:** 2016-01-21

**Authors:** Clare Holdsworth, Marina Mendonça, Hynek Pikhart, Martin Frisher, Cesar de Oliveira, Nicola Shelton

**Affiliations:** 1 School of Physical and Geographical Sciences, Keele University, Keele, UK; 2 Department of Epidemiology & Public Health, University College London, London, UK; 3 School of Pharmacy, Keele University, Keele, UK

**Keywords:** ALCOHOL, AGEING, SELF-RATED HEALTH, DEPRESSION

## Abstract

**Background:**

Older people who drink have been shown to have better health than those who do not. This might suggest that moderate drinking is beneficial for health, or, as considered here, that older people modify their drinking as their health deteriorates. The relationship between how often older adults drink and their health is considered for two heath states: self-rated health (SRH) and depressive symptoms.

**Methods:**

Data were analysed from the English Longitudinal Study of Ageing (ELSA), a prospective cohort study of older adults, using multilevel ordered logit analysis. The analysis involved 4741 participants present at wave 0, (1998/1999 and 2001), wave 4 (2008/2009) and wave 5 (2010/2011). The outcome measure was frequency of drinking in last year recorded at all three time points.

**Results:**

Older adults with fair/poor SRH at the onset of the study drank less frequently compared with adults with good SRH (p<0.05). Drinking frequency declined over time for all health statuses, though respondents with both continual fair/poor SRH and declining SRH experienced a sharper reduction in the frequency of their drinking over time compared with older adults who remained in good SRH or whose health improved. The findings were similar for depression, though the association between depressive symptoms and drinking frequency at the baseline was not significant after adjusting for confounding variables.

**Conclusions:**

The frequency of older adults’ drinking responds to changes in health status and drinking frequency in later life may be an indicator, rather than a cause, of health status.

## Introduction

The relationship between drinking and health in later life is an important component of older age mortality and morbidity. In the UK, for example, older adults consume less than younger age groups, though drink more regularly.^1^ ^2^ Statistics for mortality and hospital admissions demonstrate that health problems associated with excessive drinking are most acute in later life. Alcohol-related mortality is higher among older age groups and is increasing among the elderly while stabilising and declining at younger ages.^3^ Between 2002 and 2010 in England and Wales, the number of alcohol-related admissions into hospital for men aged 65 increased by 175% and for women by 145%.^4^

However, the causal relationship between health and drinking is complex. There is a well-established J-shaped relationship between health and alcohol, as moderate drinkers have better health outcomes than both abstainers and heavy drinkers, though the explanation for this association in disputed.^5^ In contrast to those who abstain, older moderate drinkers (as measured by quantity of consumption) have better cognition,^6^^–^8 improved well-being,^6^ and less substantial functional health decline or limitations.^9^ ^10^ Moderate drinkers also report fewer depressive symptoms, medication use and acute health events,^11^ and overall have lower mortality risks,^9^ including cardiovascular disease.^12^ At the other end of the drinking spectrum, heavy drinkers are more likely to suffer mild cognitive impairment,^7^ greater incidence of pain,^13^ higher mortality,^14^ increased risk of injury^15^ and, for men only, greater risk of depression.^16^

The possibility that there might be health benefits associated with moderate drinking has generated considerable interest and discussion. Yet the extent to which these findings can confirm the causality between alcohol consumption and health remains contested.^5^ ^17^^–^20 The relationship between abstinence and poor health can be explained partly by the ‘sick quitter effect’, so drinkers are selected for good health.^21^ Longitudinal studies have sought to control for this bias by distinguishing lifetime abstainers from former drinkers, as the latter group are potentially sick quitters.^5^ ^22^ ^23^ However, this distinction may not be sufficient to confirm causality. Studies need to consider that lifetime non-drinkers may also be selected for poor health,^24^ and healthy drinkers suffer mortality before old age.^25^

We sought to analyse how changes in health statuses (as measured by SRH and depressive symptoms) in later life were associated with changes in drinking frequency over a 10-year period. The analysis considered whether the frequency of drinking in later life is an indicator of health status and the possibility that older adults, who continue to drink frequently in later life, do so because they are healthy. Using longitudinal data on both drinking frequency and health status, the analysis considers how health is associated with drinking frequency at the onset of the period of observation and whether older adults moderate how often they drink as their health changes over time. This analysis was restricted to drinking frequency for two reasons. First, research on alcohol consumption has focused on the relationship between quantity of alcohol consumed and health outcomes. It has been shown that older adults drink more often though overall consumption declines with age.^2^ ^3^ ^26^ ^27^ However, it remains unclear how changing health status in later life affects the regularity of drinking. Second, we are not seeking to identify a dose response of alcohol consumption in response to changes in health, but consider how older adults’ drinking behaviours, as measured by frequency, change as their health deteriorates or improves.

The analysis considered two health outcomes: self-rated health (SRH) and depression. SRH was used as an indicator of health, as it is a strong predictor of mortality.^28^ The relationship between SRH and drinking confirms that good health is associated with consumption rather than abstinence.^29^ ^30^ Depression is associated with increased risk of problem drinking in later life,^16^ ^31^ and it has been shown that moderate consumption is associated with better mental health.^6^ By comparing the relationship between the two health variables and drinking frequency, we can demonstrate if different health statuses have similar influences on drinking. Variables to capture sociodemographic status and life style behaviours (wealth, employment, marital status, education, body mass index (BMI) and smoking) are included in the analysis to examine the extent to which the relationship between health outcomes and frequency of drinking can be explained by confounding variables.

## Data

**Figure 1:**
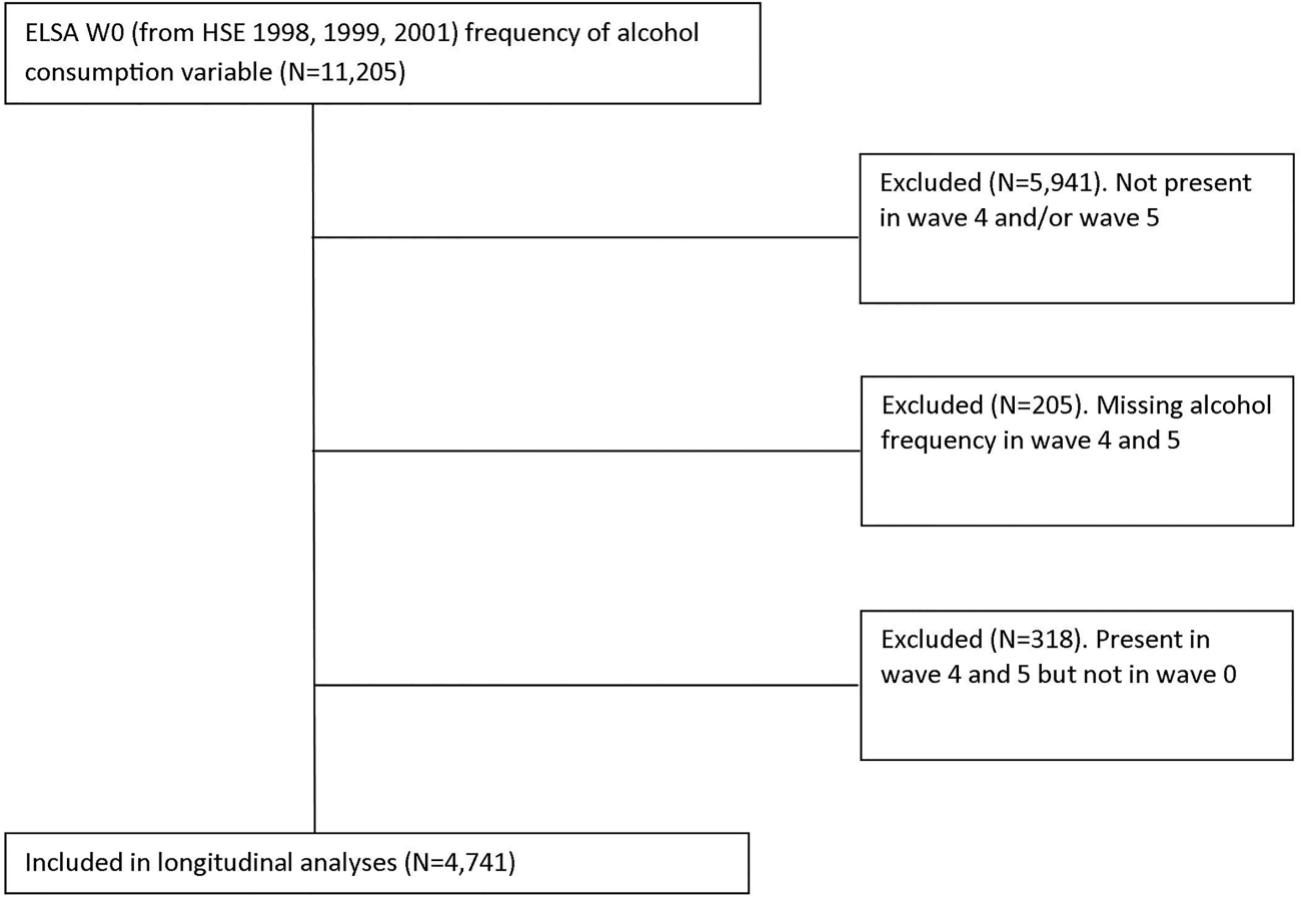
Flow chart illustrating selection of longitudinal sample (ELSA, English Longitudinal Study of Ageing; HSE, Health Survey for England).

Data obtained from waves 0, 4 and 5 of the English Longitudinal Study of Ageing (ELSA) were used for this analysis.^32^ The derivation of the sample for the analysis is outlined in figure 1. Participants in wave 0 of ELSA were recruited from the Health Survey for England (HSE) in 1998, 1999 and 2001. The first wave of ELSA (wave 1, sample size 11 205) was carried out in 2002/2003. The sample has been interviewed every 2 years, with wave 4 recruited in 2008/2009 and wave 5 in 2010/2011. Though ELSA has included questions on alcohol consumption in all waves, the precise questions asked have varied. Our analysis is restricted to waves 0, 4 and 5 where there was comparability in questions on alcohol consumption. The analysis was restricted to respondents in all three waves (4741 cases), and we excluded respondents who have either left the study over time, or were recruited to refresh it over the 10-year period.

### Measures

*Outcome variable: Frequency of alcohol consumption.* Waves 0, 4 and 5 of ELSA included a measure about the frequency of any alcohol consumed in the past 12 months. Responses varied from 1 ‘almost every day’ to 8 ‘not at all in the past 12 months’. Non-drinkers in ELSA did not answer this question, and we have combined this group with participants who did not drink at all in the past 12 months to form the category ‘does not drink’. The distribution of this variable at the three waves is given in table 1. Just over one in five older adults reported drinking every day at wave 0, though by wave 5, non-drinkers were the modal group.

**Table 1:** Frequency of drinking in last year by wave (N=4741)

Frequency of drinking	Wave 0	Wave 4	Wave 5
	Per cent	Per cent	Per cent
Does not drink	8.7	21.3	22.4
Once/twice a year	7.1	7.9	9.4
Once every couple of months	5.6	6.3	6.2
1–2 times a month	11.7	9.8	10.5
1–2 days a week	24.9	21.4	20.8
3–4 days a week	15.2	10.5	10.5
5–6 days a week	5.0	5.7	4.5
Almost every day	21.9	15.3	15.6

Base: English Longitudinal Study of Ageing (ELSA) members in waves 0, 4 and 5.

There are a small number of respondents with missing information in wave 4 (2.24%) and wave 5 (1.88%).

### Covariates

*Self-rated health*: Participants were asked to rate their health on a five-point scale. The responses were converted into a two level variable—good and fair/poor. We developed a variable to measure transitions in health which distinguished between individuals who reported stable SRH between waves 0 and 5, that is, had constant good or fair/poor SRH in both waves, and individuals whose health changed over the 10-year period, that is, their health either improved (fair/poor to good) or deteriorated (good to fair/poor) between waves 0 and 5.

*Depressive symptoms*: Depressive symptoms were measured by a short version of the Centre for Epidemiological Studies-Depression (CES-D) scale containing eight dichotomous items about depressive symptoms experienced in the last week.^33^ We derived a CES-D score by summing responses to all eight dichotomous questions. These summary scores were dichotomised using a cut point of four or higher (≥4), which is equivalent to the conventional cut point of 16 or higher on the full 20-item CES-D representing being ‘at risk’ of depression.^33^ This variable distinguishes between individuals who were in the same ‘at risk’ of depression between waves 0 and 5 and individuals who either became ‘at risk’ or stopped being ‘at risk’ over the 10-year time period between waves 0 and 5.

Other covariates (see online supplementary appendix 1) measured at wave 0 included gender, age, marital status, education, employment status, BMI and smoking behaviour. Additional information on net non-pension household wealth for each individual was obtained from wave 1 of the study. This is a summary measure of the value of financial, physical and housing wealth owned by the household minus any debt. Wealth was included in the model as a relative measure divided into quintiles. In the final models, all variables were fitted including missing cases though these are not reported in the Results section.

### Data analysis

To address the proposed aim for this study, we used a multilevel modelling approach and models were run separately for each health variable as a covariate to compare how changes in these two health statuses were associated with drinking frequency over time. Since drinking frequency over the previous year is a categorical ordered variable, rather than a linear variable, we performed a multilevel ordered logit analysis with STATA V.13, using command meologit. The longitudinal ELSA data can be viewed as having a two-level hierarchical structure as follows. Level 1 described within-person change; that is, how respondents’ frequency of drinking changed over time. Level 2 described between-person differences in drinking frequency over time. Analysis at this level provides information about the individuals’ initial drinking frequency (intercept) and trajectory during the study period (slope). In this analysis, we investigated how the rate of change over time in frequency varied according to changes in two health statuses (SRH and depression). Data on the frequency of drinking were available at three time points (waves 0, 4 and 5), and the measure of time in the analysis was wave which was fitted as a categorical variable with three categories. Level 2 analysis was included by fitting an interaction term between health status and wave.

## Results

Tables 2 and 3 summarise the relationship between the transition variables for SRH and depressive symptoms and the frequency of drinking at wave 0. This cross-sectional analysis demonstrated a clear relationship between good health/no depression and frequent drinking. One-quarter of all adults who reported good health drank every day at wave 0, compared with 14.8% of those with fair/poor health. Moreover, in wave 0, adults whose health/depression symptoms subsequently deteriorated were more likely to drink infrequently or not drink at all. Among older adults whose SRH deteriorated, 18.5% drank a few times a year or not at all at wave 0 compared with 10.8% of those who remained in good health at all subsequent waves; for depression, the equivalent percentages were 18.6% and 13.4%.

**Table 2:** Cross-tabulation of frequency of drinking at wave 0 by transitions in self-rated health between waves 0 and 5

Frequency of drinking wave 0	Self-rated health: transitions between waves 0 and 5			
	Good health wave 0		Fair/poor health wave 0	
	Continual good health n=2753 Per cent	Health deteriorates n=748 Per cent	Health remains fair/poor n=596 Per cent	Health improves n=497 Per cent
Does not drink	5.4	9.1	16.8	13.9
Once/twice a year	5.4	9.4	11.0	9.5
Once every couple of months	5.4	6.7	6.6	6.4
1–2 times a month	11.7	10.7	11.9	13.3
1–2 days a week	26.1	25.5	22.1	22.3
3–4 days a week	16.4	12.1	13.1	13.7
5–6 days a week	5.8	4.5	3.7	3.6
Almost every day	24.6	22.0	14.8	17.3

Pearson χ^2^=208.0838, p<0.05.

**Table 3:** Cross-tabulation of frequency of drinking at wave 0 by transitions in depressive symptoms between waves 0 and 5

Frequency of drinking wave 0	Depressive symptoms: transitions between waves 0 and 5			
	No depressive symptoms wave 0		Depressive symptoms wave 0	
	Continual no depression n=3328 Per cent	Becomes depressed n=279 Per cent	Depression remains n=473 Per cent	Depression improves n=386 Per cent
Does not drink	7.4	10.8	14.3	10.6
Once/twice a year	6.0	7.8	12.9	11.4
Once every couple of months	4.9	8.0	7.2	9.3
1–2 times a month	11.3	12.7	13.3	13.2
1–2 days a week	25.9	24.7	19.4	23.3
3–4 days a week	15.6	13.5	12.9	13.2
5–6 days a week	5.9	3.0	2.5	2.3
Almost every day	23.1	19.4	17.6	16.6

Pearson χ^2^=107.7379, p<0.05.

Analysis of key variables has shown that attrition between waves 0 and 4/5 was higher among non-drinkers rather than drinkers.^34^ This was confirmed using logistic regression analysis of being present in wave 5 for wave 0 respondents. This analysis found that non-drinkers in wave 0 were less likely to be present in wave 5 compared with all other drinking frequencies. To consider the possible impact of attrition on the findings, the longitudinal models were carried out for younger cohort of older adults (age less than 70 in wave 0) who were less likely to leave the study over the 10-year period. This analysis of this restricted age cohort gave the same results as the full models which are reported below, suggesting that attrition did not affect the results.

The results of the longitudinal analysis are given in table 4 (for analysis including transitions in SRH) and table 5 (for analysis including transitions in depressive symptoms). We report the ordered log-odds regression coefficients for two models for each health variable: model 1a included transitions in SRH, gender, age at wave 0 and transition in SRH×wave interaction and model 1b included these variables and all covariates measured at wave 0: employment status, education, marital status, smoking, BMI and wealth (measured at wave 1). Models 2a and 2b replicated this analysis substituting transitions in depressive symptoms for SRH. Negative coefficients indicate that individuals with a particular characteristic had a higher chance of drinking less frequently compared with those in the reference category; positive coefficients indicate a higher chance of more frequent drinking.

**Table 4:** Ordered logit model of drinking frequency of ELSA participants in waves 0, 4 and 5 controlling for transitions in self-rated health: N=4741

Variable	Model 1a		Model 1b		Variable	Model 1b	
	Coefficient	p Value	Coefficient	p Value		Coefficient	p Value
Transitions in self-rated health×wave					Marital status wave 0		
Wave 0					Married	0.00	
Continual good health	0.00		0.00		Single	−0.28	0.25
Health deteriorates	−0.48	0.01	−0.11	0.52	Separated/divorced	0.13	0.45
Continual poor health	−1.63	<0.01	−0.99	<0.01	Widowed	0.04	0.80
Health improves	−1.27	<0.01	−0.93	<0.01	Employment wave 0		
Wave 4					Employed	0.00	
Continual good health	0.00		0.00		Economically inactive	<0.01	0.99
Health deteriorates	−1.09	<0.01	−0.81	<0.01	Retired	0.12	0.43
Continual poor health	−2.29	<0.01	−1.81	<0.01	Wealth wave 1		
Health improves	−1.47	<0.01	−1.20	<0.01	Bottom quintile	0.00	
Wave 5					Second quintile	0.73	<0.01
Continual good health	0.00		0.00		Third quintile	1.00	<0.01
Health deteriorates	−1.09	<0.01	−0.81	<0.01	Fourth quintile	1.61	<0.01
Continual poor health	−2.41	<0.01	−1.99	<0.01	Top quintile	2.34	<0.01
Health improves	−1.55	<0.01	−1.31	<0.01	Education wave 0		
Continual good health					No education	0.00	
Wave 0	0.00		0.00		Compulsory education	0.63	<0.01
Wave 4	−0.81	<0.01	−0.90	<0.01	Postcompulsory Education	0.80	<0.01
Wave 5	−0.90	<0.01	−1.00	<0.01	Degree or higher	1.75	<0.01
Health deteriorates between waves 0 and 5					Smoking wave 0		
Wave 0	0.00		0.00		Non-smoker	0.00	
Wave 4	−1.41	<0.01	−1.60	<0.01	Former occasional smoker	0.38	0.08
Wave 5	−1.65	<0.01	−1.89	<0.01	Former regular smoker	1.27	<0.01
Continual poor health					Current smoker	0.81	<0.01
Wave 0	0.00		0.00		BMI wave 0		
Wave 4	−1.47	<0.01	−1.72	<0.01	25–30	0.00	
Wave 5	−1.68	<0.01	−1.99	<0.01	<20	−0.62	0.11
Health improves between waves 0 and 5					20–25	0.28	0.03
Wave 0	0.00		0.00		>30	−0.51	<0.01
Wave 4	−1.02	<0.01	−1.16	<0.01			
Wave 5	−1.18	<0.01	−1.37	<0.01			
Gender ref: Male							
Female	−1.54	<0.01	−1.18	<0.01			
Age at wave 0 (confounding variable)	−0.04	<0.01	−0.03	<0.01			
Likelihood ratio test*		<0.01		<0.01			

Model 1a includes SRH×wave, age wave 0 and gender.

Model 1b includes SRH×wave, age wave 0, gender, marital status, employment, net household wealth, education, smoking and BMI.

*The likelihood ratio test compares the meologit model with an ologit model, that is, if the mixed-effects ordered logit regression model is favoured over a standard ordered logit regression model.

BMI, body mass index; ELSA, English Longitudinal Study of Ageing; SRH, self-rated health.

**Table 5:** Ordered logit models of drinking frequency of ELSA participants in waves 0, 4 and 5 controlling for transitions in depressive symptoms: N=4741

Variable	Model 2a		Model 2b		Variable	Model 2b	
	Coefficient	p Value	Coefficient	p Value		Coefficient	p Value
Transitions in depressive symptoms×wave					Marital status wave 0		
Wave 0					Married	0.00	
No depression	0.00		0.00		Single	−0.27	0.26
Becomes at risk of depression	−0.52	0.01	−0.08	0.67	Separated/divorced	0.10	0.54
Continual depression	−1.04	<0.01	−0.14	0.56	Widowed	0.08	0.62
Depression improves	−0.98	<0.01	−0.34	0.09	Employment wave 0		
Wave 4					Employed	0.00	
No depression	0.00		0.00		Economically inactive	−0.31	0.05
Becomes at risk of depression	−1.18	<0.01	−0.72	<0.01	Retired	−0.03	0.85
Continual depression	−2.20	<0.01	−1.27	<0.01	Wealth wave 1		
Depression improves	−1.46	<0.01	−0.82	<0.01	Bottom quintile	0.00	
Wave 5					Second quintile	0.73	<0.01
No depression	0.00		0.00		Third quintile	1.09	<0.01
Becomes at risk of depression	−1.18	<0.01	−0.72	<0.01	Fourth quintile	1.60	<0.01
Continual depression	−1.94	<0.01	−1.02	<0.01	Top quintile	2.47	<0.01
Depression improves	−1.24	<0.01	−0.60	0.02	Education wave 0		
No depression					No education	0.00	
Wave 0	0.00		0.00		Compulsory education	0.68	<0.01
Wave 4	−0.96	<0.01	−0.95	<0.01	Postcompulsory education	0.87	<0.01
Wave 5	−1.14	<0.01	−1.13	<0.01	Degree or higher	1.86	<0.01
Becomes at risk of depression between waves 0 and 5					Smoking wave 0		
Wave 0	0.00		0.00		Non-smoker	0.00	
Wave 4	−1.61	<0.01	−1.59	<0.01	Used to smoke occasionally	0.35	<0.01
Wave 5	−1.73	<0.01	−1.71	<0.01	Used to smoke regularly	1.20	<0.01
Continual depression					Current smoker	0.67	<0.01
Wave 0	0.00		0.00		BMI wave 0		
Wave 4	−2.12	<0.01	−2.08	<0.01	25–30	0.00	
Wave 5	−2.04	<0.01	−2.01	<0.01	<20	−0.60	0.13
Depression improves between waves 0 and 5					20–25	0.34	0.01
Wave 0	0.00		0.00		>30	−0.61	<0.01
Wave 4	−1.43	<0.01	−1.42	<0.01			
Wave 5	−1.39	<0.01	−1.39	<0.01			
Gender							
Ref: male	0.00		0.00				
Female	−1.55	<0.01	−1.18	<0.01			
Age at wave 0 (confounding variable)	−0.04	<0.01	−0.03				
Likelihood ratio test*		<0.01		<0.01			

Model 2a includes depressive symptoms×wave, age wave 0 and gender.

Model 2b includes depressive symptoms×wave, age wave 0, gender, marital status, employment, net household wealth, education, smoking and BMI.

*The likelihood ratio test compares the meologit model with an ologit model, that is, if the mixed-effects ordered logit regression model is favoured over a standard ordered logit regression model.

BMI, body mass index; ELSA, English Longitudinal Study of Ageing.

Age and gender were negative and significant in all models, illustrating that older women drank less frequently than men and for both genders drinking frequency declined with age. The results for the different health variables were broadly similar, though there was a small difference between the unadjusted and adjusted models for depressive symptoms compared with SRH. Taking SRH first (models 1a and 1b), there was a strong association between SRH and the frequency of drinking. In the unadjusted model (1a), respondents with continual fair/poor, improving and deteriorating SRH had a higher chance of drinking less frequently compared with respondents in continual good health at all waves. The regression coefficients were greatest for the category continual fair/poor SRH, demonstrating that this group were more likely to drink less frequently than all other health categories at all waves. The coefficients for each health status at waves 4 and 5 illustrate that all adults, regardless of health status, were more likely to drink less frequently in later waves. These coefficients were greater for older adults in continual fair/poor or declining SRH, compared with those with continual good or improving health. Thus, the differential in drinking frequency by SRH observed at wave 0 increased over time. The coefficients for the non-adjusted and adjusted models were broadly similar; though including confounding variables in model 1b reduced the differences in drinking frequency by health status at each wave, but enhanced the difference within each health status over time. The differential at wave 0 between older adults in continual good health and those whose health subsequently declined was no longer significant in model 1b.

The findings for depressive symptoms were broadly similar to SRH in the unadjusted model (2a). However, after covariates were included in the adjusted model (2b), the association between drinking frequency and depressive symptoms at wave 0 was not significant. Yet in subsequent waves, and in both unadjusted and adjusted models, older adults with continual depressive symptoms were more likely to drink less frequently at all waves. The decline over time for this group was greater than other categories of depressive symptoms. As with SRH older adults whose depressive symptoms improved over time experienced a smaller decline in frequency compared with those with continual depressive symptoms and who became at risk of depression. Older adults with no risk of depression at all waves drank more often than all other categories and experienced the smallest decline in frequency between the waves.

## Discussion

Older adults with fair/poor SRH throughout the period of observation and those whose SRH deteriorated over the 10 years experienced a faster decline in the frequency of drinking compared with older adults with constant good SRH. Respondents whose SRH improved over the period drank less frequently over time, but this reduction was less compared with older adults whose SRH deteriorated or remained poor/fair. Thus, the selection effect of SRH on drinking frequency is shown to be ongoing and older adults with continual good or improving SRH reported more consistent drinking over time.^30^ The relationship between depressive symptoms and drinking frequency is broadly similar to SRH. The relationship between drinking and both health variables is modified in the adjusted models, which demonstrates that socioeconomic characteristics explain some of observed relationship between health and drinking frequency, particularly for depressive symptoms.

The strength of this study is that it considers the dynamic relationship between two health statuses, SRH and depressive symptoms, and drinking frequency over time. The analysis demonstrates that alterations in drinking frequency in later life were associated with health conditions and how these changed over time. We confirm earlier studies that found declining frequency of drinking in later life associated with health, gender, wealth, education and age.^29^ ^30^ ^35^^–^37 The finding that drinking frequency declines over time among older adults contrasts with analysis of trends in drinking by age that suggest that adults drink more frequently as they get older, though overall consumption declines with age.^2^ ^3^ These results extend the findings of previous analysis through considering the relationship between changes in health and drinking frequency. Our findings support the suggestion that frequent drinking in later life signifies good health, measured by SRH and depressive symptoms.^17^ For depression, existing research demonstrates that depression is a risk factor for heavy drinking.^16^ ^31^ The results of this analysis demonstrate that having symptoms of depression is associated with less frequent drinking. This suggests that some older people with depression may self-medicate with alcohol and consume more, while others may reduce how often they drink, especially given adverse interactions with medication.

However, it should be noted that the analysis of the longitudinal cohort data cannot fully account for attrition from ELSA between each wave. Attrition between waves 0 and 4/5 was higher among non-drinkers rather than drinkers. This has implications for the analysis of the relationship between health and drinking frequency, if those present in wave 5 exhibited a different relationship between health and drinking, compared with older adults who were not present in all waves. The analysis is also restricted to health outcomes for which there are sufficient data to carry out the longitudinal analysis. For this reason, the analysis is restricted to more generalised health status (SRH) and depressive symptoms which has a high occurrence among ELSA participants (25% of the sample was at risk of depression in at least one wave).

In summary, the analysis shows that older people with continual good SRH and mental health drink more often and their frequency of drinking is more consistent over time. Thus, disentangling a causal relationship between drinking and health is more complex than simply distinguishing between life-long abstainers and former drinkers. We concur with others that there is insufficient evidence to endorse the recommendation that drinking is beneficial for health.^17^ There are other confounding factors that impact on drinking in later life and health, and it is not possible to infer about possible health benefits in isolation of other individual characteristics and behaviours. In particular, more affluent older adults drink more and this group also enjoys better health,^38^ and analysis of the potential health benefits of drinking needs to carefully control for this association.

Though the analysis cannot confirm a causal relationship between health and frequency of drinking, on balance we conclude that the evidence suggests that we have to take seriously the suggestion that frequent drinking in later life is a signifier of good health. However, while the importance of the sick quitter effect is well established, relatively little is known about the mechanisms through which health status may moderate drinking behaviours. Further research could explore why older people drink less as their health declines, including the role of medical advice, possible interactions with medication and fewer opportunities to socialise due to deteriorating health.

**What is already known on this subject**

- Consumption of alcohol among older people in England has remained stable in recent years in contrast to declining consumption among younger age groups. Alcohol-related mortality and morbidity is also increasing among the individuals over 55 years. The putative relationship between good health and moderate alcohol consumption is increasingly being contested.

**What this study adds**

- This study provides evidence that regular alcohol consumption in later life is an indicator of good health rather than a cause of good health. The message that moderate drinking is beneficial in later life should be reviewed.

## Supplementary Material

Web supplement
