# Supplementary material for: Is regular drinking in later life an indicator of good health? Evidence from the English Longitudinal Study of Ageing
Source: J Epidemiol Community Health. 2016 Jan 21;70(8):764–70. doi: 10.1136/jech-2015-206949 (PMC4975801; doi:10.1136/jech-2015-206949)
Supplement: Web supplement [file jech-2015-206949-s1.pdf]

Appendix 1: Distribution of Variables (n=4741)

Variable	Number of participants in all 3 waves (%)	Variable	Number of participants in all 3 waves (%)
**Health**		**Wealth**	
Good health all waves	2753 (58.1%)	Bottom Quintile	620 (13.1%)
Poor health all waves	748 (15.8%)	2nd Quintile	860 (18.1%)
Health deteriorates between waves	596 (12.6%)	3rd Quintile	955 (20.1%)
Health improves between waves	497 (10.5%)	4th Quintile	1059 (22.3%)
Other/missing	147 (3.1%)	Top Quintile	1168 (24.6%)
**Depression**		Missing	79 (1.7%)
No depression all waves	3328 (70.1%)	**Education**	
Becomes depressed between waves	279 (5.9%)	No education	1744 (36.8%)
Depression in all waves	473 (10.0%)	Compulsory education	1466 (30.9%)
Depression improves between waves	386 (8.1%)	Post compulsory education	925 (19.5%)
Other/missing	275 (5.8%)	Degree or higher	638 (13.5%)
**Gender**		Missing/not known	1 (0.0%)
Male	2048 (43.2%)	**Smoking**	
Female	2693 (56.8%)	Non smoker	1856 (39.2%)
**Marital Status Wave 0**		Used to smoke occasionally	300 (6.3%)
Married	3303 (69.7%)	Used to smoke regularly	1802 (38.0%)
Single	236 (5.0%)	Current smoker	781 (16.5%)
Separated/divorced	544 (11.5%)	Missing	2 (0.0%)
Widowed	655 (13.8%)	**BMI**	
Other/missing	3 (0.0%)	25-30	85 (1.8%)
**Employment Wave 0**		<20	1172 (24.7%)
Employed	2063 (43.5%)	20-25	2054 (43.3%)
Economically inactive	770 (16.2%)	>30	1087 (22.9%)
Retired	1904 (40.2%)	Missing	343 (7.2%)
Other/missing	4 (0.1%)		
